# Impact Behavior of Composite Reinforced Concrete Beams with Pultruded I-GFRP Beam

**DOI:** 10.3390/ma15020441

**Published:** 2022-01-07

**Authors:** Teghreed H. Ibrahim, Abbas A. Allawi, Ayman El-Zohairy

**Affiliations:** 1 Department of Civil Engineering, University of Baghdad, Baghdad 17001, Iraq; tagreed.hassan@coeng.uobaghdad.edu.iq (T.H.I.); A.Allawi@uobaghdad.edu.iq (A.A.A.); 2 Department of Engineering and Technology, Texas A&M University-Commerce, Commerce, TX 75429, USA

**Keywords:** pultruded GFRP I-beam, composite beam, impact, static, experiments, deflections, finite element analysis

## Abstract

The present study experimentally and numerically investigated the impact behavior of composite reinforced concrete (RC) beams with the pultruded I-GFRP and I-steel beams. Eight specimens of two groups were cast in different configurations. The first group consisted of four specimens and was tested under static load to provide reference results for the second group. The four specimens in the second group were tested first under impact loading and then static loading to determine the residual static strengths of the impacted specimens. The test variables considered the type of encased I-section (steel and GFRP), presence of shear connectors, and drop height during impact tests. A mass of 42.5 kg was dropped on the top surface at the mid-span of the tested beams from five different heights: 250, 500, 1000, 1500, and 1900 mm. Moreover, nonlinear Finite Element (FE) models were developed and validated using the experimental data. Static loading was defined as a displacement-controlled loading and the impact loading was modeled as dynamic explicit analysis with different drop velocities. The validated models were used to conduct a parametric study to investigate the effect of the concrete compressive strength on the performance of the composite beams under static and impact loadings. For the composite specimen with steel I-sction, the maximum impact force was 190% greater than the reference specimen NR-I at a drop height of 1900 mm, whereas the maximum impact forces for the specimens composite specimens with GFRP I-sction without and with shear connectors were 19% and 77%, respectively, more significant than the reference beam at the same drop height. The high stiffness for the steel I-beams relative to the GFRP I-beam was the reason for this difference in behavior. The concrete compressive strength was more effective in improving the impact behavior of the composite specimens relative to those without GFRP I-beams.

## 1. Introduction

Hybrid construction using combined materials is essential for achieving performance targets such as durability, sustainability, seismic resistance, and speedy construction. Because composite materials are simple, buildable, and cost-competitive, they give a consistent performance and are the best choice. Pultruded Glass Fiber Reinforced polymer (GFRP) I-beams are a new type of Fiber Reinforced Polymer (FRP) composite that are developed using pultrusion. These new beams are recommended for bridge construction due to their superior corrosion resistance and higher compressive and flexural strength [1,2]. The behavior of concrete beams under the effect of impact loading is different than under the effect of static loading [3]. The materials are subjected to a higher strain rate when loaded dynamically than statically. There is little work in the literature on the impact and load rate sensitivity of pultruded composite materials. Understanding the behavior of such materials is becoming increasingly important as the use of these unconventional materials for infrastructure applications is developed.

Previous studies explored different types of composite beams under the effect of impact loading. Tabiei et al. [4] investigated the influence of loading velocity on the GFRP beam section of a square box without concrete to determine the critical design parameters. Static and impact tests at different impact velocities were performed to determine the loading rate sensitivity of the pultruded box beams. The ultimate load decreased as the impact velocity increased. The impact behavior of GFRP pultruded rectangular hollow profile filled with concrete was investigated experimentally by Li et al. [5]. The hollow box profile provided tensile strength and protection for the internal concrete block from chemical attacks and the filled concrete provided the system with structural stability. Soleimani and Roudsari [6] studied the impact behavior of reinforced concrete (RC) beams with and without externally bounded sprayed and fabric GFRP under impact and quasi-static loading. A mass of 591 kg was dropped with various heights and loading rates. The stiffness of the tested beams decreased with increasing the drop height. Moreover, applying sprayed GFRP (with and without mechanical stiffeners) and fabric GFRP on the surface of RC beams increased the stiffness. The concrete strength affected the bending characteristics under impact loading [7]. It increased the maximum impact load by 59% and decreased the damping ratio by 47% when the compressive strength increased from 20 MPa to 50 MPa.

Nonlinear Finite Element (FE) models were developed and calibrated to analyze the impact behavior of composite RC beams with pultruded GFRP materials [5,6,7,8]. Analytical models were developed using Abaqus to analyze structural members strengthened with GFRP sheets and subjected to different dynamic loading conditions (quasi-static, impact) [8]. The developed models could be an effective tool to predict the performance of retrofitted beams under dynamic loading conditions. Furthermore, it showed that FRP retrofitting RC beams subjected to repetitive impact loads could effectively improve their dynamic performance and slow damage progress. Abaqus used the load-time curves of the experimental study to check parameters such as deflection, strain, and stresses as a function of time for analysis. A hollow solution was used to solve the equation set to identify the unknown variable (this method was included in the Abaqus program) [7].

Composite RC beams with pultruded GFRP beams are widely used for bridge construction due to their superior corrosion resistance and higher compressive and flexural strength [1,2]. However, the research on the impact performance of these types of beams is still very limited. Moreover, comparisons between the impact flexural properties of encased steel and GFRP I-sections as well as the effect of using shear connectors on the composite interaction need in-depth investigation. Therefore, the present study experimentally and numerically investigated the impact behavior of these beams. Eight specimens of two groups were cast in different configurations. The first group consisted of four specimens and was tested under static load to provide reference results for the second group. The four specimens in the second group were tested first under impact loading and then static loading to determine the residual static strengths of the impacted specimens. The test variables considered the type of encased I-section (steel and GFRP), presence of shear connectors, and drop height during impact tests. A mass of 42.5 kg was dropped on the top surface at the mid-span of the tested beams from five different heights: 250, 500, 1000, 1500, and 1900 mm. Moreover, nonlinear Finite Element (FE) models were developed and validated using the experimental data. Static loading was defined as a displacement-controlled loading and the impact loading was modeled as dynamic explicit analysis with different drop velocities. The validated models were used to conduct a parametric study to investigate the effect of the concrete compressive strength on the performance of the composite beams under static and impact loadings.

## 2. Experimental Program

Eight specimens of two groups were cast in different configurations, as listed in Table 1. The first group consisted of four specimens, which were tested under static load to provide reference results for the second group. The four specimens in the second group were tested first under impact loads and then static loads to determine the residual static strengths of the impacted specimens.

### 2.1. Details of Specimens

**Figure 1:**
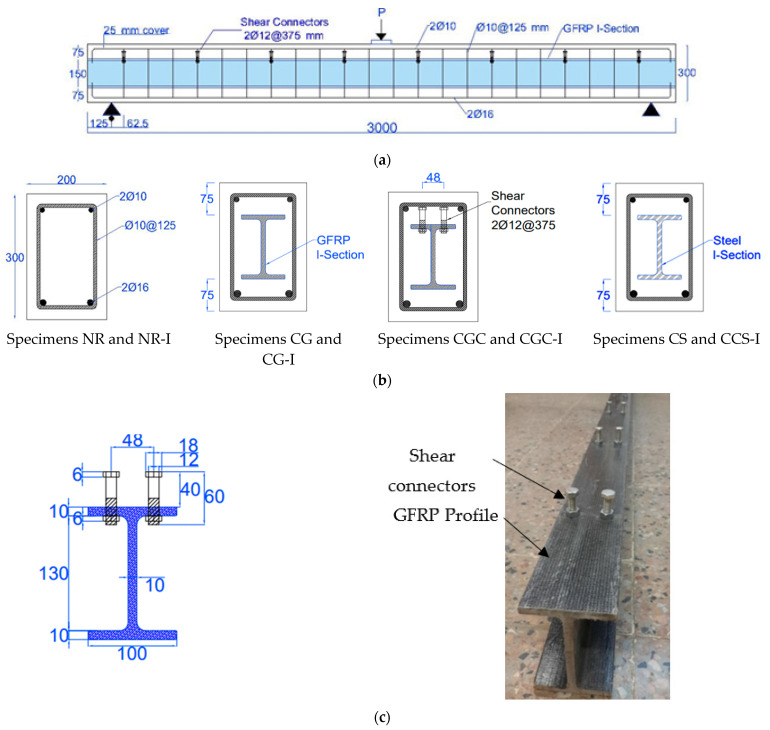
Details of the tested specimens: (**a**) Elevation of the specimens; (**b**) Cross -sections of the tested specimens; (**c**) Geometrical dimensions of the GFRP profile and installment of the shear connectors (Unit: mm).

The overall length of the specimens was 3000 mm with a clear span of 2750 mm, as shown in Figure 1a. All specimens had the same cross-sectional dimensions with a width of 200 mm and a total thickness of 300 mm, as illustrated in Figure 1b. Conventional reinforcements were used for the reference specimens NR and NR-I. The flexural reinforcements were two rebars with a diameter of 16 mm in tension and two rebars with a diameter of 10 mm in compression. The transverse was stirrups with a diameter of 10 mm diameter at a spacing of 125 mm to prevent premature shear failure. The flexural and shear reinforcement were the same for the other specimens. Four specimens CG, CG-I, CGC, and CGC-I were reinforced with pultruded GFRP I-beams positioned at the centroid of the cross-section as shown in Figure 1b. Dimensions of the pultruded GFRP I-sections are illustrated in Figure 1c. Shear connectors were provided at the top flange of the GFRP I-section in specimens CGC and CGC-I to increase the composite interaction between the GFRP I-beam and concrete. The diameter of these connectors was 12 mm with a height of 70 mm and a spacing of 375 mm. The shear connectors were stiffened with washers and nuts after being inserted through drilled holes on both sides of the top flange of the GFRP I-beam. The remaining two specimens CS and CS-I were reinforced with steel I-beams positioned at the center of the cross-section as shown in Figure 1b.

### 2.2. Material Properties

Concrete specimens were prepared and tested to determine the compressive strength, splitting tensile strength, modulus of rupture, and modulus of elasticity of concrete. Three standard cubes of dimensions 150 mm × 150 mm × 150 mm and three standard cylinders of dimensions 150 mm × 300 mm were tested by a universal compression machine to determine the compressive strength of concrete. The splitting tensile strength of concrete was determined according to ASTM C496-96 [9]. The modulus of elasticity of concrete was calculated according to ASTM C469 [10] by using standard concrete cylinders 150 mm × 300 mm. The mechanical properties of concrete are listed in Table 2.

The mechanical properties of steel bars and steel plates, such as yield tensile strength and ultimate tensile strength, were evaluated according to ASTM A370-19 [11]. Three steel bars with a diameter of ∅ 10 mm and ∅ 16 mm with 0.5 m length and two steel plates with flat coupons were tested. The results are listed in Table 3.

The mechanical properties of the GFRP I-section, such as compressive and tensile strength and elastic modulus, were examined. The compression tests were conducted following ASTM D695 [12]. Fifteen specimens with a coupon dimension of 10 mm × 12.7 mm × 38.1 mm were tested. Ten coupons were cut from the longitudinal direction of the flange and the web of the I-section and five coupons were cut from the web in the transverse direction. Tension tests were conducted following ISO 527 [13] on ten specimens with coupon dimensions of 10 mm × 25 mm × 250 mm. All coupons were cut from the longitudinal direction, five from the flange and five from the web. The average mechanical properties of the tested coupons are listed in Table 3. Moreover, the longitudinal and transverse modulus of elasticity of the GFRP beam were 27.1 GPa and 6.8 GPa, respectively, as provided by the manufacturer (DURA composites, Clacton On Sea, UK).

### 2.3. Instrumentation and Test Setup

#### 2.3.1. Static Test

**Figure 2:**
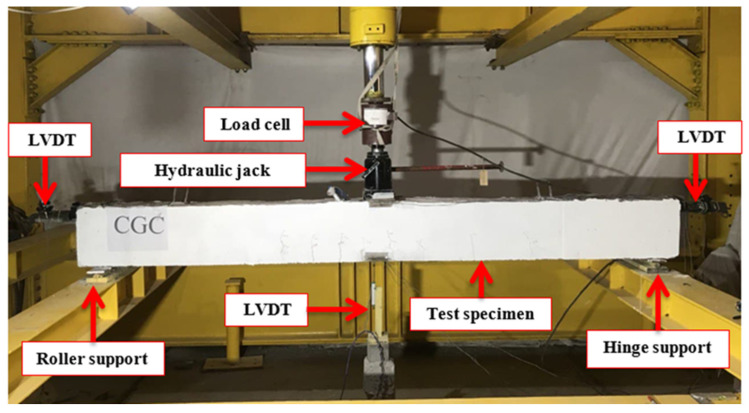
Test setup for static tests.

Beam specimens of group one were tested as simply-supported beams under a monotonic concentrated load at the mid-span up to failure using a 1000 kN capacity hydraulic universal testing machine, as shown in Figure 2. The applied load was monitored using a load cell with a 1000 kN capacity. Moreover, a linear variable differential transformer (LVDT) was used to measure the vertical deflection at the mid-span of the tested specimen.

#### 2.3.2. Impact Test

**Figure 3:**
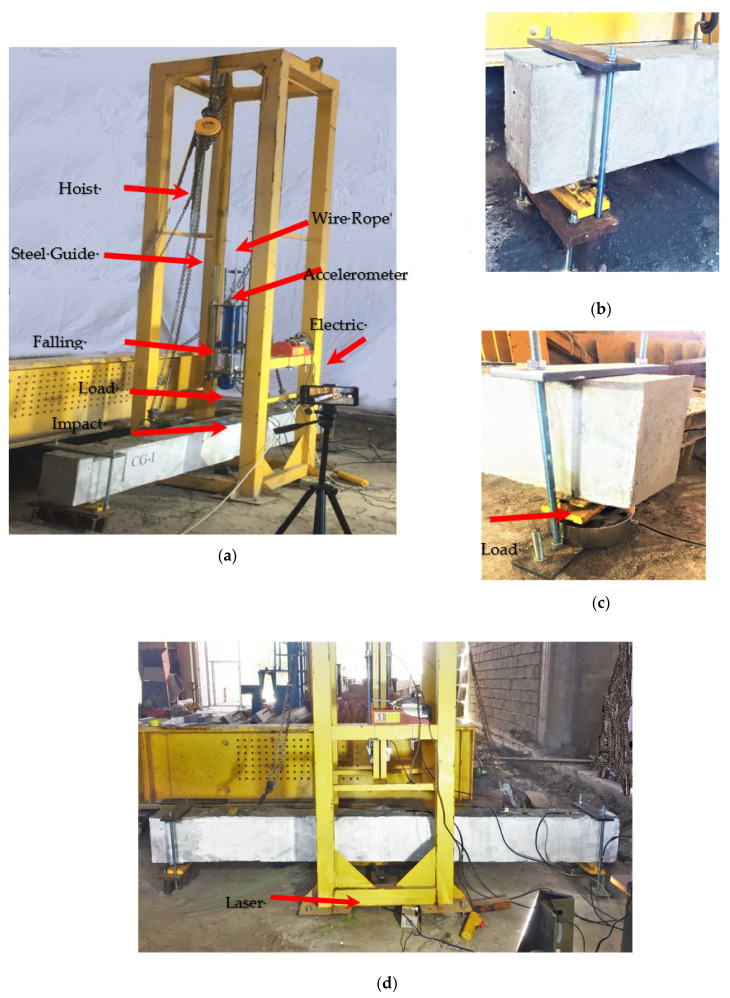
Setup of the impact test: (**a**) Steel frame of the impact test; (**b**) Left support with steel yokes; (**c**) Right support with load cell; (**d**) Front view of the test.

Figure 3 illustrates the loading frame of impact tests performed with a drop mass impactor. The mass of 42.5 kg was dropped on the top surface at the mid-span of the tested beams from five different heights: 250, 500, 1000, 1500, and 1900 mm. A hoist and chain system was used to raise the hammer over the specimen during testing. The dropped mass was manually released according to the specified height and steel guide rails were used to vertically drop the mass. 

The hammer has the potential energy $m_{h}$ × $a_{h}$× H (mass of the hammer × acceleration of the hammer under gravity × height with respect to the specimen’s top surface). When the hummer was raised up and released, the potential energy was converted to kinetic energy as the hammer fell with an acceleration $a_{h}$. Due to the frictional forces of the machine, the downward acceleration of the hammer was less than the acceleration of gravity (9.81 m/s^2^). Just before the hammer struck the beam, the velocity and kinetic energy were calculated by Equations (1) and (2), respectively.

(1) $$v=\sqrt{2a_{h}H}$$

(2) $$Ek=\frac{1}{2}m_{h}\cdot v^{2}=m_{h}\cdot a_{h}\cdot H$$

When the hammer hit the beam, the hammer’s momentum was rapidly transferred to the beam. As a response, the hammer’s momentum was reduced. This resulted in a reduction in the hammer’s kinetic energy and an increase in the beam’s energy. This sudden energy transfer between the hammer and the beam led to a rapid build-up of stress in the beam. The momentum (M) is obtained from Equation (3). The impact characteristics of the impact tests are listed in Table 4.

(3) M=m·v

The specimens were tested at a clear span of 2750 mm. The setup of the two supports allowed for rotation while preventing twisting of the specimens. The vertical movement of supports was restrained using two steel yokes (see Figure 3b,c). The impact loads were recorded while striking the specimen using two load cells. A dynamic load cell with a capacity of (300 kN) was rigidly connected to the drop mass system between the steel shaft and the impactor (Figure 3a). The second load cell with a capacity of 1000 kN was placed under one support to measure the reaction under support (see Figure 3c). The mid-span deflection was measured for each stepped time using a laser velocity sensor from Keyence Company LK-081 (Itasca, IL, USA) with the controller LK-2101 (Itasca, Illinois, U.S.A). The measuring range of the laser velocity sensor is ± 15mm, as provided by the manufacturer. In order to measure the impactor velocity, an accelerometer was attached to the upper surface of the impactor. All output results were collected by a data recorder of type DATAQ DI-710 (Dataq Instruments Inc., Akron, OH, USA). After conducting the impact tests, the residual load-carrying capacity of the beam specimens was determined using static loading tests until failure.

## 3. Experimental Results

### 3.1. Static Test Results

#### 3.1.1. Crack Patterns and Modes of Failure

**Figure 4:**
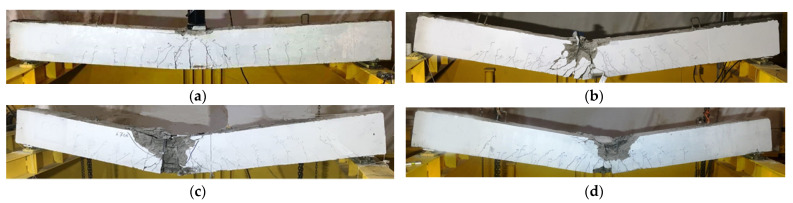
Modes of failure of the tested specimens under static test: (**a**) Specimen NR; (**b**) Specimen CG; (**c**) Specimen CGC; (**d**) Specimen CS.

The first crack was flexural cracking and formed at the mid-spans of the tested specimens. The cracking loads of the composite specimens CG, CGC, and CS were 4%, 5%, and 53% higher than that one for the reference specimen NR, respectively. Then, more cracks were created and propagated from the loading point along the specimen’s longitudinal axis. Shear cracks appeared at the final stage of loading. The failure mechanism of the reference specimen NR was yielding in the tension steel rebars followed by crushing in concrete in the compression zone as shown in Figure 4a. For composite specimens CG and CGC and after reaching the ultimate loads, concrete in the compression zones was crushed, buckling of the compression steel rebars and cover spalling occurred, initial inter-laminar failure and rupture of the GFRP profile was accompanied by a loud noise as described in Figure 4b,c. For composite specimen, CS with steel I-beam, crushing of concrete in the compression zone started at the load of 140 kN. After that, a plastic region was created in the steel I-beam, where the deflection increased rapidly while the applied load ranged from 160 to 170 kN (see Figure 4d).

#### 3.1.2. Load-Deflection Behavior

**Figure 5:**
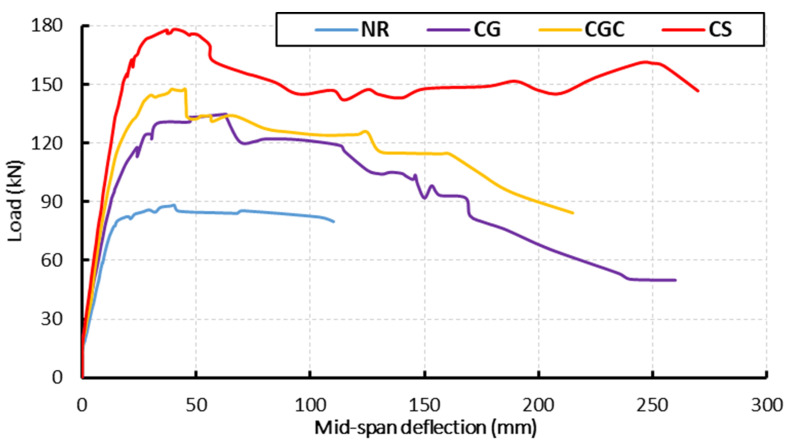
Load-deflection relationships for the tested specimens under static loads.

Figure 5 illustrates the load-deformation relationships of the tested specimens. The specimens showed linear relationships between the applied loads and deflections until the yielding of the steel reinforcement. For composite specimens with GFRP I-beam and steel I-beam, the yielding loads increased by 38%, 51%, and 104% for specimens CG, CGC, and CS relative to the reference specimen NR, respectively. Moreover, the stiffness was enhanced for these specimens. Table 5 lists a summary of the experimental results. The ultimate capacities of the composite specimens CG and CGC were 134.62 kN and 147.23 kN, respectively. The embedded GFRP I-beam in these specimens increased the load-bearing capacity by 51% and 65%, respectively. Increasing the composite interaction between the GFRP I-beam and concrete by means of shear connectors improved the ultimate capacity by 9.5% relative to the composite specimen without shear connectors. For specimen CS with steel I-beam, the steel reinforcement in tension yielded at a load of 162.5 kN, which is 87.5% higher than that one for the reference specimen NR. Subsequently, the load increased to the ultimate value of 178.3 kN, which is 100% higher than that for the reference specimen NR. Then the applied load decreased by 10% due to the yielding of the steel I-beam at the mid-span.

### 3.2. Impact Test Results

#### 3.2.1. Crack Patterns and Modes of Failure

**Figure 6:**
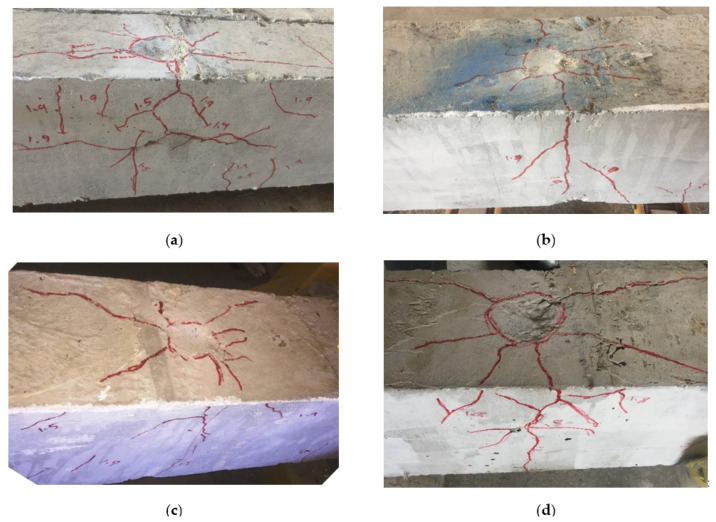
Local damage at the impact zone of the impact tested specimens: (**a**) NR-I; (**b**) CG-I; (**c**) CGC-I; (**d**) CS-I.

**Figure 7:**
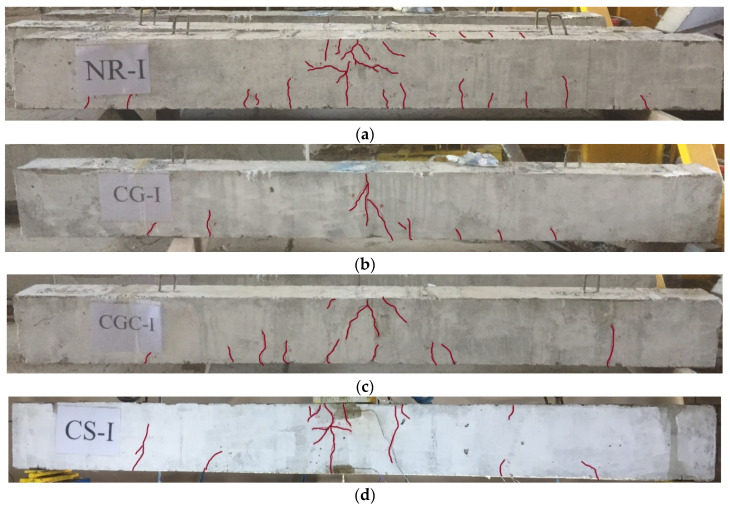
Crack patterns on the side face of the impacted specimens: (**a**) NR-I; (**b**) CG-I; (**c**) CGC-I; (**d**) CS-I.

Cracks were initially formed at the point of impact on the top surfaces of the tested specimens followed by visible flexural cracks at the beam soffit as shown in Figure 6. For the NR-I specimen, cracks initially appeared when the mass hit the beam from a height of 1.0 m followed by flexural cracks, which spread along the beam. However, the shear cracks appeared when the mass hit the beam from a height of 1.5 m. Moreover, spalling of the concrete occurred at the impact zone and cracks on the proximal face of concrete caused penetration of 8 mm with an 82.5 mm boring diameter (see Figure 6a). For CG-I and CGC-I specimens, cracks occurred at the middle area of the specimen when the mass hit the beam from a height of 1.5 m. However, the flexural cracks propagated down the beam and appeared when the height was 1.9 m. These specimens experienced penetrations of 3 mm and 4.5 mm and boring diameters of 57.5 mm and 67.5 mm, respectively (see Figure 6b,c). For specimen CS-I, cracks began to appear after striking from a height of 1.5 m. Moreover, cracks appeared at the locality of the strike and spread to the top of the specimen with a total penetration of 17 mm and a 93.2 mm boring diameter (see Figure 6d). The crack patterns on the side faces of the specimens are illustrated in Figure 7.

#### 3.2.2. Impact Response

**Figure 8:**
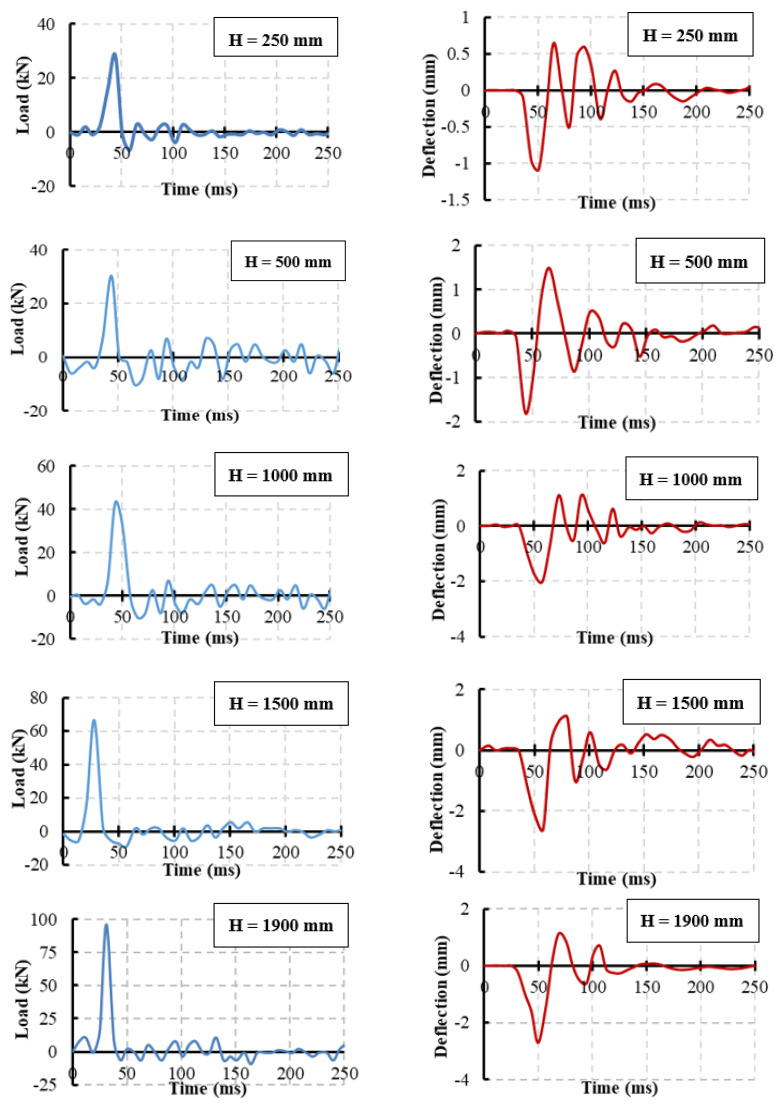
The specimen NR-I’s impact force and mid-span deflection time histories.

**Figure 9:**
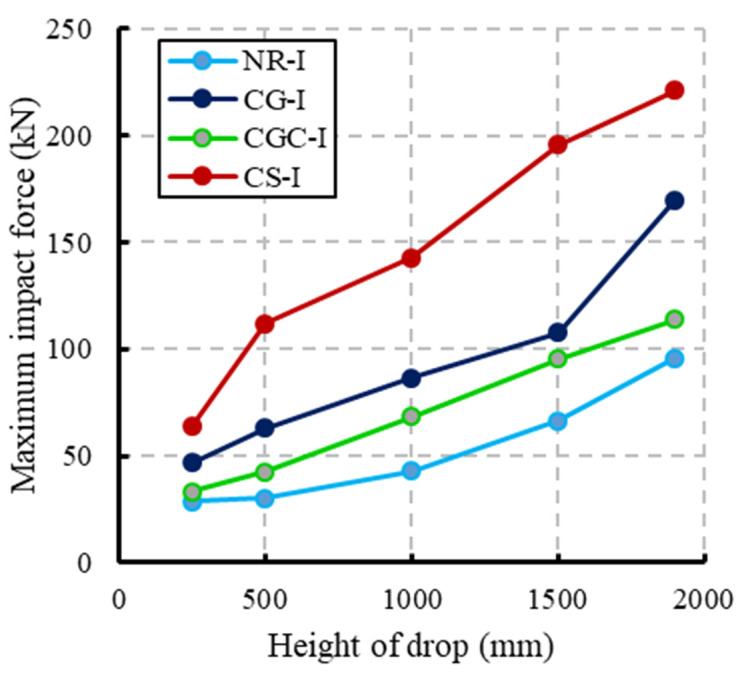
Comparisons of the maximum impact force for different heights of drop.

**Figure 10:**
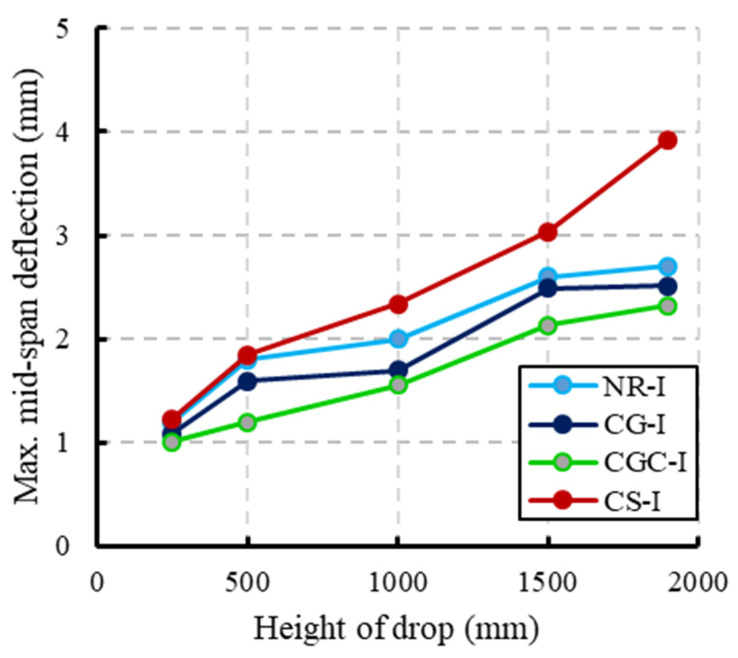
Comparisons of the maximum mid-span deflection for different heights of drop.

The impact load and deflection time histories for each specimen were recorded for each height. Figure 8 illustrates the specimen NR-I’s impact force and mid-span deflection time histories as an example of the impact response. The first pulse’s peak grew in proportion to the drop height, whereas the initial pulse-like waveform had a duration of about 25 ms regardless of the drop height. The length of the blunt waveform grew as the drop height increased, but the peaks of the blunt waveforms were approximated independently of the drop height. The maximum mid-span deflection increased as the drop height increased. Comparisons of the maximum impact force and maximum mid-span deflection of specimens NR-I, CG-I, CGC-I, and CS-I for different heights are listed in Table 6 and shown in Figure 9 and Figure 10, respectively.

Figure 9 shows that the maximum impact force increased as the drop height increased. For the composite specimen CS-I, the maximum impact force was 130% greater than the reference specimen NR-I at a drop height of 1900 mm whereas the maximum impact forces for specimens CG-I and CGC-I were 19% and 77%, respectively, more significant than the reference beam at the same drop height. The high stiffness for the steel I-beams relative to the GFRP I-beams was the reason for this difference in behavior, where the impact force is proportional to the structural stiffness. As seen in Figure 10, the composite specimens CG-I and CGC-I showed reductions in the corresponding mid-span deflections relative to the reference specimen NR-I. For the 1900 mm drop height, reductions of 10.7% and 14.1% were obtained, respectively, whereas the composite specimen CS-I showed a 45% increase in the corresponding mid-span deflection due to the high impact force caused by the section’s high stiffness.

#### 3.2.3. Residual Static Test after the Impact

**Figure 11:**
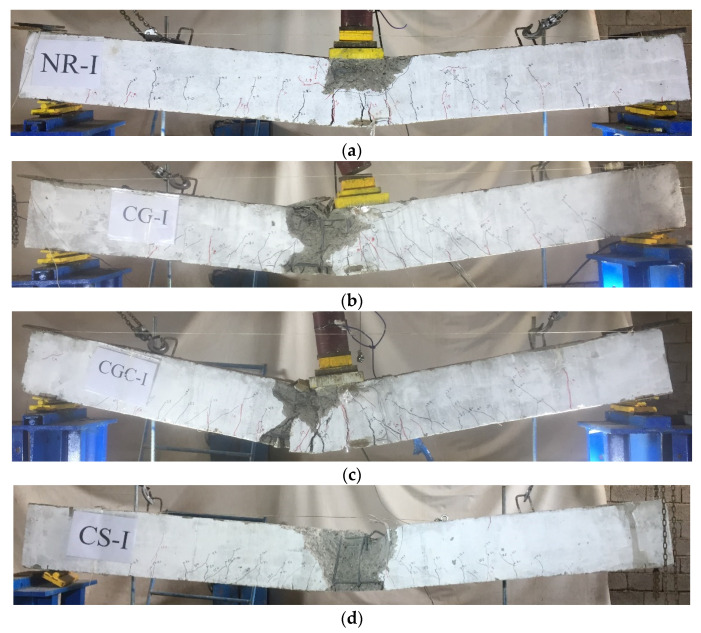
Crack patterns for impact specimens after the residual static tests: (**a**) Specimen NR-I; (**b**) Specimen CG-I; (**c**) Specimen CGC-I; (**d**) Specimen CS-I.

**Figure 12:**
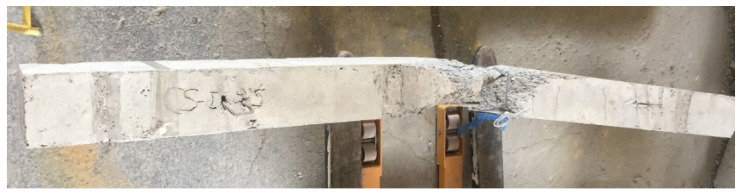
Lateral buckling failure of specimen CS-I (top view).

The residual static strengths were explored by conducting static tests until failure after the completion of the impact tests. The ultimate capacities are compared in Table 7 between the specimens without and with previous impact loads. More reduction in the reference specimen’s capacity occurred and the specimen with the GFRP I-beam without shear connectors showed the least reduction. Providing shear connectors to specimen CGC-I led to higher stiffness and significant damage relative to the specimen without shear connectors CG, which caused more reduction in the residual beam strength (see Table 7). Figure 11 shows the crack patterns of the impacted specimens after the residual static tests. Additional cracks were formed and propagated vertically and diagonally, which increased in width and depth until failure. All specimens failed in flexural mode. The specimen CS-I exhibited lateral buckling due to the high level of the applied load, which decreased the steel I-beam strength as illustrated in Figure 12.

## 4. Numerical Modeling

### 4.1. Finite Element Model

Finite element analysis was performed using the Abaqus software [14]. The developed model considered two groups of loading conditions. The first group was the static loading defined in terms of general static procedures. The loading was applied as displacement-controlled loadings at the center of the beam whereas the second group was the repeated impact loading determined as dynamic explicit analysis. The loading was applied using the impactor at the mid-spans of the beams as initial velocities. The proposed models were validated using the experimental test results.

The concrete, shear connectors, and steel I-profile were modeled using three-dimensional eight-node elements with reduced integration (C3D8R). The longitudinal and transverse steel reinforcement were modeled using three-dimensional truss elements (T3D2) with a linear kinematic hardening model. The pultruded GFRP I-profile was simulated using an 8-node doubly curved thick shell element with reduced integration (S8R). The impactor in the impact loading case was modeled by a discrete rigid shell that rotated 360° to create the cylindrical shape.

### 4.2. Material Models

**Figure 13:**
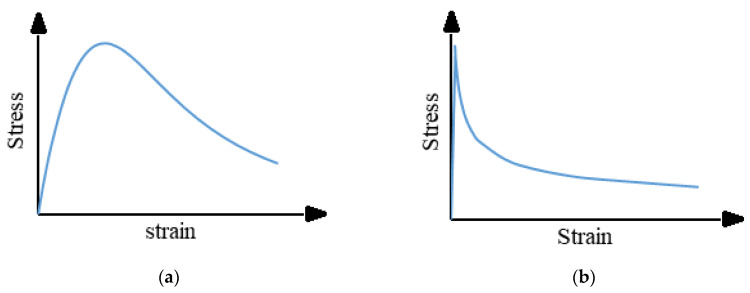
Stress-strain relationships for concrete [16]. (**a**) Compressive stress-strain curve; (**b**) Tensile stress-strain curve.

The concrete damage plasticity (CDP) model was employed to model the behavior of the tested specimens to consider the concrete cracking and crushing. The response of the CDP model under uniaxial tensile loading was characterized by a linear–elastic stress-strain relationship up to the value of the failure stress. The stress-strain curves were used to describe the compressive and tensile behaviors, as shown in Figure 13 [15]. Table 8 lists the different damage parameters for cracking and crushing. These parameters included the dilation angle (φ), eccentricity (ε), compressive strength to uniaxial pressure ratio biaxial (f_bo_⁄f_co_), coefficient (K), and viscosity parameters (μ). These parameters were established from previous analyses and were implemented in this study.

**Figure 14:**
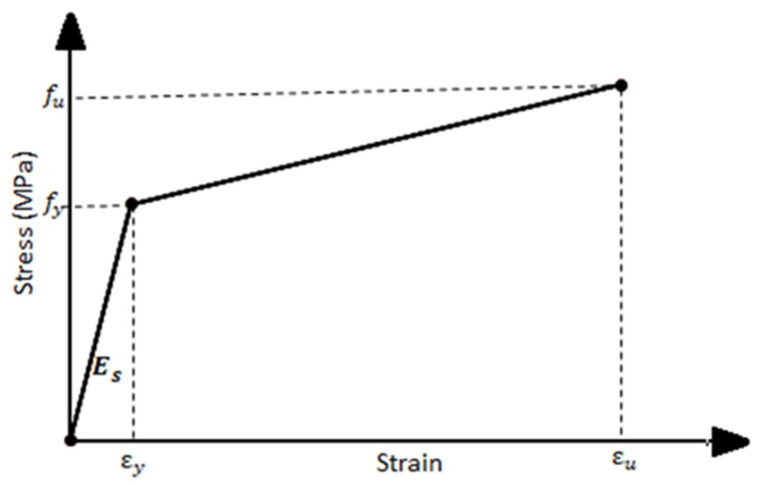
Stress-strain relationship of steel I-beam, rebars, and stirrups.

The steel I-beam, longitudinal steel rebars, and stirrups were modeled as a non-linear relationship adopted by [15], as shown in Figure 14. The linear isotropic part is defined by the modulus of elasticity of the reinforcement and Poisson’s ratio. The plastic part is defined by the yield stress f_y_, ultimate stress f_u_, and plastic strains, as illustrated in Figure 14. Using a combination of damage initiation criteria, the progressive damage model was employed to characterize the behavior of the pultruded profiles. Hashin’s criteria [17] were used to determine when the pultruded profile begins to degrade. After satisfying the damage initiation criteria, the material stiffness of the pultruded profile degrades according to the damage evolution law. Table 9 lists the values of engineering constants for elastic properties of GFRP material, the strength properties to represent the damage intuition criteria, and the parameters for the progressive damage model presented by damage evolution (fracture energy) and damage stabilization indicated by viscosity coefficients. The values in this table were determined by experimental tests and from the available works of literature [16,18] based on the type of fibers and matrix as well as trial and error.

### 4.3. Static Analysis

**Figure 15:**
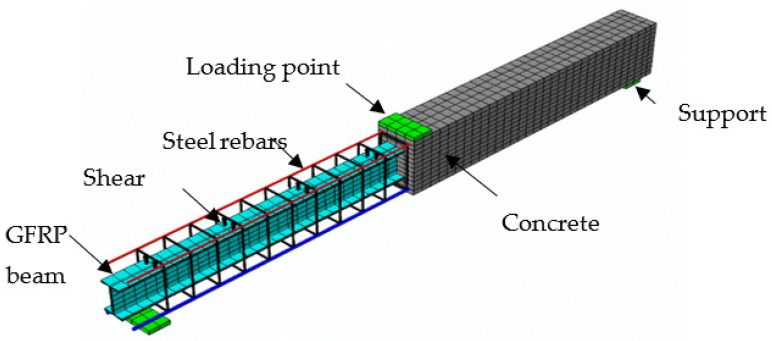
FE mesh of the analyzed specimen CGC for static analysis.

The numerical model employed the general static processes by using the displacement-controlled loading. The suggested model was validated using experimental data from the tested specimens NR, CG, and CGC. The assembled FE model for the composite specimen CGC is shown in Figure 15. The embedded technique with a complete bond was employed to simulate the interaction between the steel rebars and concrete as well as the GFRP profile and concrete.

#### Model Verifications

**Figure 16:**
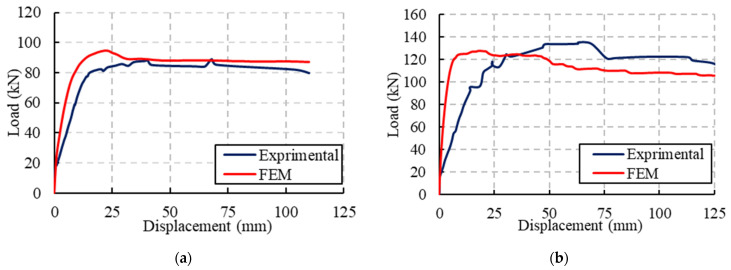

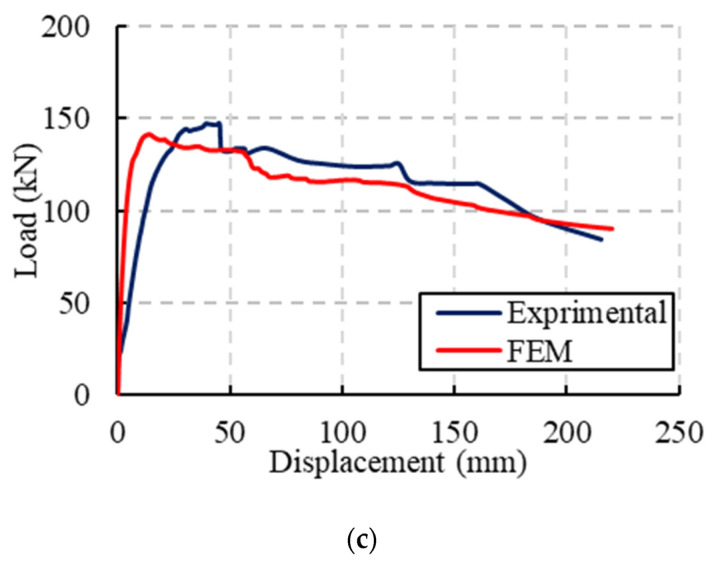
Validations of the FE results for static loading: (**a**) Specimen NR; (**b**) Specimen CG; (**c**) Specimen CGC.

Figure 16 presents comparisons between the experimental and FE results in terms of the load-deflection relationships. The analyzed beams showed initially linear elastic behavior with a higher stiffness than the experimental results. These beams showed ±6% or less high capacities relative to the experimental results. The perfect bond assumed between the concrete and steel rebars as well as the GFRP profile was the main reason for this difference in response. Moreover, the boundary conditions, as well as the material constitutive models, were considered ideal in the numerical analyses relative to those in the experiments. In general, there was good agreement between the experimental and FE results under static loading.

### 4.4. Impact Analysis

**Figure 17:**
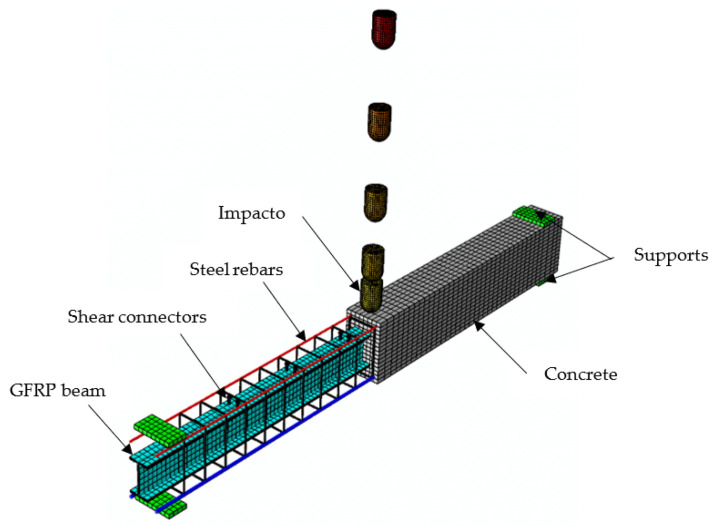
FE mesh of the composite specimen CGC-I for impact analysis.

The numerical model employed the dynamic explicit step to apply the impact loading. The suggested model was validated using the experimental data of specimens NR-I, CG-I, and CGC-I. FE models were developed to represent five series impacts by five identical impactors that hit the model at different drop velocities. The contact between the hammer and top surface of the beam was employed as surface-to-surface contact, which used a friction coefficient of 0.35 with tangential behavior. Figure 17 shows the assembled FE model for the composite specimen CGC. The time period used was 100 ms for each hit. During the repeated blows, the mid-span deflection and impact force were recorded for each hit.

#### Model Verifications

**Figure 18:**
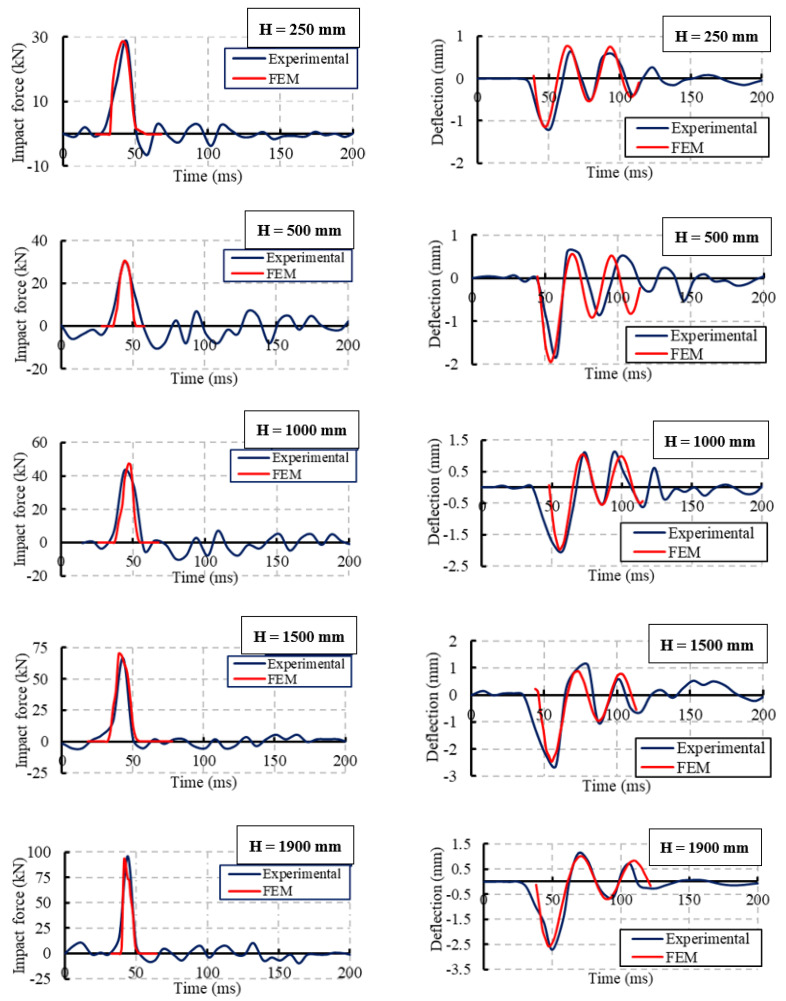
Validations of the experimental and FE results of the impact force and mid-span deflection time histories for specimen NR-I.

Comparisons between the FE and experimental results of the impact force and mid-span deflection time histories for specimen NR-I, as an example, are shown in Figure 18. The numerical simulations and experiment results were validated using five impact hits. Good agreement between the experimental and FE results was obtained. The difference between the maximum impact loads in the FE model and the experimental result was 10% of all specimens. Moreover, this difference was about 6% of the mid-span deflections for all specimens.

## 5. Parametric Study

A parametric study was carried out to investigate the influence of the concrete compressive strength on the ultimate strength of the composite specimens with the GFRP I-beams under the effect of static and impact loadings.

### 5.1. Static Analysis

**Figure 19:**
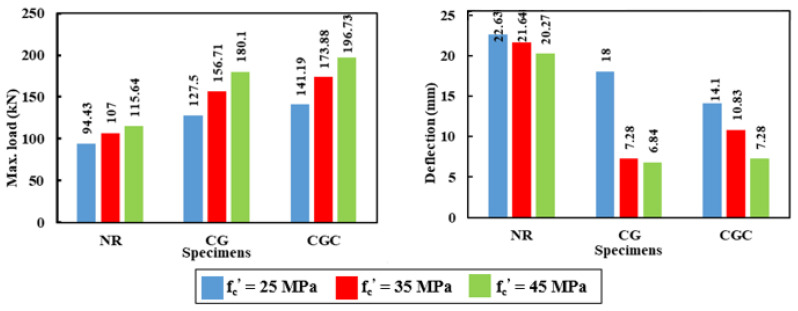
The effect of concrete compressive strength on the ultimate load capacity and corresponding deflection for the analyzed specimens.

The influence of different concrete compressive strengths of 25 MPa, 35 MPa, and 45 MPa on the static flexural behavior of the analyzed specimens was investigated. Table 10 and Figure 19 illustrate the concrete compressive strength effect on the ultimate load capacity and the corresponding deflection. As the compressive strength increased, the ultimate load capacity increased and the corresponding deflection decreased. The increasing and decreasing percentages were calculated based on the 25 MPa as the reference compressive strength. For the composite specimens, the ultimate load capacity was improved by 23% and 40% for compressive strengths 35 MPa and 45 MPa, respectively. However, these improvements were 13% and 22% for the reference specimen NR, respectively. On the other hand, the corresponding deflections were reduced by 60% for the specimen CG. However, these reductions were 23% and 48% for the concrete strength 35 MPa and 45 MPa, respectively, for the specimen CGC. These results confirmed the effectiveness of concrete strength to improve the stiffness of the composite specimens is less pronounced when the shear connectors were used. However, the same effect on the ultimate load capacity was obtained. For specimen NR, the corresponding mid-span deflections were reduced only by 4.4% and 10.4% for the concrete strengths 35 MPa and 45 MPa, respectively. The concrete compressive strength was more effective in improving the behavior of the composite specimens relative to those without GFRP I-beams.

### 5.2. Impact Analysis

**Figure 20:**
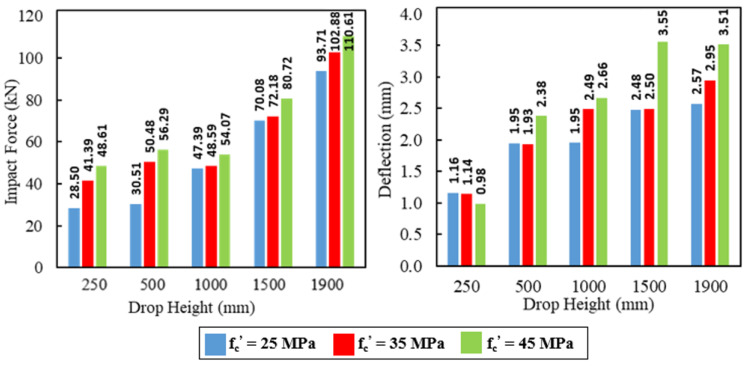
The effect of the concrete compressive strength on the impact forces and mid-span deflections for the reference specimen NR-I.

**Figure 21:**
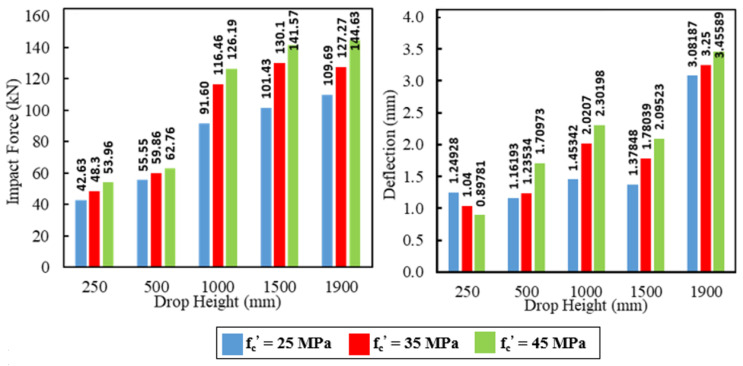
The effect of concrete compressive strength on the impact forces and mid-span deflections for the composite specimen CG-I.

**Figure 22:**
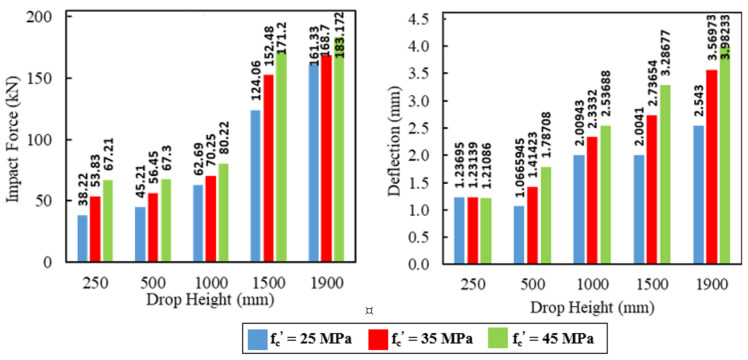
The effect of concrete compressive strength on the impact forces and mid-span deflections for the composite specimen CGC-I.

The concrete compressive strengths 25 MPa, 35 MPa, and 45 MPa were examined to determine their influences on the load capacity and corresponding mid-span deflection under the effect of impact loads. Figure 20, Figure 21 and Figure 22 show these influences for the five impact hits. Relative reductions were noticed in the deflections at the maximum impact load for the first hit (H = 250 mm), whereas increases were observed for the other hits. The first hit had a more negligible effect on the behavior of the analyzed model. Therefore, the deflection in the first hit had a behavior similar to that of deflection in the static loading.

Variation percentages in the impact load and mid-span deflection were calculated based on the results of the compressive strength 25 MPa as listed in Table 11 and Table 12, respectively. The average increase in the maximum impact load was between 18% and 25% for all specimens when the compressive strength was 35 MPa. However, it was between 30% and 40% when the compressive strength was 45 MPa. Likewise, for deflection, the average difference in the maximum deflection at the maximum impact load was between 9% and 25% for all specimens when the compressive strength was 35 MPa. This difference was ranged between 30% and 43% when the compressive strength was 45 MPa. The impact characteristics of the RC composite specimens with GFRP I-beam were influenced effectively by increasing the compressive strength of concrete.

## 6. Conclusions

The present study experimentally and numerically investigated the impact behavior of composite RC beams with the pultruded I-GFRP and I-steel beams. Eight specimens of two groups were cast in different configurations. The first group consisted of four specimens and was tested under static load to provide reference results for the second group. The four specimens in the second group were tested first under impact loading and then static loading to determine the residual static strengths of the impacted specimens. Moreover, nonlinear FE models were developed and validated using the experimental data. The validated models were used to conduct a parametric study to investigate the effect of the concrete compressive strength on the performance of the composite beams under static and impact loadings. The main conclusions are summarized as follows:

1. The embedded GFRP I-beam in the RC specimens with and without shear connectors increased the load-bearing capacity by 51% and 65%, respectively, relative to the reference specimen NR. However, this improvement was 100% in the case of using steel I-beam.
2. Increasing the composite interaction between the GFRP I-beam and concrete by means of shear connectors improved the ultimate capacity by 9.5% relative to the composite specimen without shear connectors.
3. For the composite specimen CS-I, the maximum impact force was 190% greater than the reference specimen NR-I at a drop height of 1900 mm whereas the maximum impact forces for specimens CG-I and CGC-I were 19% and 77%, respectively, more significant than the reference beam at the same drop height. The high stiffness for the steel I-beams relative to the GFRP I-beams was the reason for this difference in behavior, where the impact force is proportional to the structural stiffness.
4. The composite specimens CG-I and CGC-I showed reductions in the maximum mid-span deflections relative to the reference specimen NR-I. For the 1900 mm drop height, reductions of 9.4% and 21% were obtained, respectively, whereas the composite specimen CS-I showed a 17% increase in the maximum mid-span deflection due to the high impact force caused by the section’s high stiffness.
5. As the compressive strength increased, the static ultimate load capacity increased and the corresponding deflection decreased. For the composite specimens with GFRP I-beam, the ultimate load capacity was improved by 23% and 40% for compressive strengths 35 MPa and 45 MPa, respectively. However, these improvements were 13% and 22% for the reference specimen NR, respectively. On the other hand, the corresponding deflections were reduced by 60% for the specimen CG. However, these reductions were 23% and 48% for the concrete strength 35 MPa and 45 MPa, respectively, for the specimen CGC. These results confirmed the effectiveness of concrete strength to improve the stiffness of the composite specimens was less pronounced when the shear connectors were used.
6. The impact characteristics of the RC composite specimens with GFRP I-beam were influenced effectively by increasing the compressive strength of concrete. The average increase in the maximum impact load was between 18% and 25% for all specimens when the compressive strength was 35 MPa. However, it was between 30% and 40% when the compressive strength was 45 MPa. Therefore, the concrete compressive strength was more effective in improving the impact behavior of the composite specimens relative to those without GFRP I-beams.

It is worth noting that these impact tests were performed to initially unloaded specimens while in reality, the composite RC beams would be loaded and then an impact load would be applied. Therefore, pre-loading before administering impact loads will be the next research focus.

## Figures and Tables

**Table 1:** Details of the tested specimens.

Group	Specimens	Cross-Section (mm)	Specimen Weight (kg/m)	Type of Test	Type of Encased Beam	Connection *
I	NR	200 × 300	503.89	Static	N/A	NSC
	CG	200 × 300	509.83	Static	GFRP I-beam	NSC
	CGC	200 × 300	510.51	Static	GFRP I-beam	SC
	CS	200 × 300	529.8	Static	Steel I-beam	NSC
II	NR-I	200 × 300	503.89	Impact and Static	N/A	NSC
	CG-I	200 × 300	509.83	Impact and Static	GFRP I-beam	NSC
	CGC-I	200 × 300	510.51	Impact and Static	GFRP I-beam	SC
	CS-I	200 × 300	529.8	Impact and Static	Steel I-beam	NSC

* NSC: No shear connectors, SC: with shear connectors.

**Table 2:** Mechanical properties of concrete.

Slump Test (mm)	Compressive Strength * (MPa)	Compressive Strength ** (MPa)	Splitting Tensile Strength (MPa)	Modulus of Rupture (MPa)	Modulus of Elasticity (MPa)
100	23.43	29.29	2.46	2.41	20,754.64

* Concrete cylinders, ** Concrete cubes.

**Table 3:** Mechanical properties of steel reinforcement and GFRP.

Material	Diameter (mm)	Thickness (mm)	Yield Strength (MPa)	Transverse Compressive Strength (MPa)	Longitudinal Compressive Strength (MPa)	Longitudinal Tensile Strength (MPa)	Longitudinal Modulus of Elasticity (GPa)	Transverse Modulus of Elasticity (GPa)
Steel	16	-	520.73	-	-	687.07	200	-
	10	-	407.7	-	-	465.63	200	-
	-	10	375.9	-	-	479.63	200	-
GFRP	-	10	-	118.3	326.14	347.5	27.1	6.8

**Table 4:** Characteristics of the impact test.

Specimens	Striker Mass (kg)	Drop Height (m)	Tested Acceleration of Striker m/s^2^	Impact Velocity (m/s)	Kinetic Energy (Joule)	Momentum (kg·m/s)
NR-I	42	0.25	7.63	1.95	80.09	82.02
	42	0.5	6.31	2.51	132.53	105.51
	42	1.0	6.72	3.67	282.15	153.95
	42	1.5	6.55	4.43	412.66	186.18
	42	1.9	4.89	4.31	389.93	180.98
CG-I	42	0.25	6.56	1.81	68.91	76.08
	42	0.5	6.96	2.64	146.25	110.84
	42	1.0	5.45	3.30	228.84	138.64
	42	1.5	5.32	3.99	335.11	167.78
	42	1.9	5.86	4.72	467.28	198.12
CGC-I	42	0.25	8.38	2.05	87.96	85.96
	42	0.5	7.01	2.65	147.11	111.16
	42	1.0	7.51	3.87	315.32	162.75
	42	1.5	5.86	4.19	369.28	176.12
	42	1.9	8.26	5.60	659.38	235.35
CS-I	42	0.25	6.14	1.75	64.50	73.61
	42	0.5	7.57	2.75	158.94	115.55
	42	1.0	8.12	4.03	340.99	169.24
	42	1.5	6.68	4.48	420.94	188.04
	42	1.9	6.22	4.86	496.47	204.21

**Table 5:** Summary of the experimental results under static tests.

Specimen	Crack Load P_cr_ (kN)	Yield Load P_y_ (kN)	Ultimate Load P_u_ (kN)	Increase in Ultimate Load (%)	Mode of Failure
NR	19.82	79.5	89.17	-	Yield to steel bars and concrete crushing.
CG	20.62	110.4	134.62	51	Delamination and transverse shear failure in the web of the GFRP I-section.
CGC	20.89	120.2	147.23	65	Delamination and transverse shear failure in the web of the GFRP I-section.
CS	30.03	162.5	178.3	100	Yielding of steel I-section, crushing of compressive concrete.

**Table 6:** Impact test results.

Specimen	Height of Drop (mm)	Impact Force (kN)	% Change	Corresponding Mid-Span Deflection (mm)	% Change
NR-I	250	28.52	-	1.20	-
	500	30.21	-	1.80	-
	1000	43.02	-	2.00	-
	1500	66.53	-	2.60	-
	1900	95.82	-	2.70	-
CG-I	250	46.79	+64%	1.09	−9.1%
	500	62.83	+30%	1.59	−11.6%
	1000	86.58	+100%	1.95	−2.5%
	1500	95.32	+43%	2.39	−8.1%
	1900	113.98	+19%	2.41	−10.7%
CGC-I	250	33.25	+17%	1.01	−15.8
	500	42.70	+41%	1.20	−33.3%
	1000	68.57	+59%	1.95	−2.5%
	1500	107.56	+62%	2.13	−18.1%
	1900	169.79	+77%	2.32	−14.1
CS-I	250	63.72	+123%	1.23	2.5%
	500	111.96	+270%	2.1	16.1%
	1000	142.81	+232%	2.34	17.2%
	1500	195.61	+194%	3.04	16.9%
	1900	221.24	+130%	3.92	45.1%

**Table 7:** Ultimate capacities of the specimens without and with previous impact loads.

Specimens	Without Impact Loads (kN)	With Impact Loads (kN)	Residual Static Strength (%) *
NR/NR-I	89.17	75.2	84
CG/CG-I	134.62	133.1	99
CGC/CGC-I	147.23	137.5	93
CS/CS-I	178.3	163.3	91

* Percentage of the static strength of specimens without impact loading.

**Table 8:** Input parameters for the CDP model.

Parameter	φ	Eccentricity ε	*f_bo_⁄f_co_*	K	μ
Value	31°	0.1	1.16	0.667	0.001

**Table 9:** GFRP materials properties and progressive damage parameters input in ABAQUS.

	Definition	Value
Engineering Elastic Constants	Longitudinal Modulus of Elasticity (E_z_)E	27.1 GPa **
	Transverse Modulus of Elasticity (E_x_ = E_y_)	6.8 GPa
	Transverse Shear Modulus of Elasticity (G_xy_)	17.5 GPa
	In-Plane Shear Modulus of Elasticity (G_zx_ = G_zy_)	2.7 GPa
	Major Poisson’ Ratio (υ_zx_ = υ_zy_)	0.23
	Minor Poisson’ Ratio (υ_xy_)	0.1
Strength Values	Longitudinal Tensile Strength	347.5 MPa
	Longitudinal Compressive Strength	326.14 MPa
	Transverse Tensile Strength	50 MPa
	Transverse Compressive Strength	118.3 MPa
	Transverse Shear Strength	8.04 MPa
	In-Plane Shear Strength	104.23 MPa
Damage Evolution	Longitudinal Tensile Fracture Energy	18.3
	Longitudinal Compressive Fracture Energy	5.8
	Transverse Tensile Fracture Energy	5
	Transverse Compressive Fracture Energy	5.5
Damage Stabilization	Viscosity Coefficient in the Longitudinal Tensile Direction	0.001
	Viscosity Coefficient in the Longitudinal Compressive Direction	0.001
	Viscosity Coefficient in the Transverse Tensile Direction	0.001
	Viscosity Coefficient in the Transverse Compressive Direction	0.001

**Table 10:** Summary of the concrete compressive strength effects.

Specimens	Compressive Strength (MPa)	Max. Load (kN)	Increase in Max. Load (%)	Deflection at Max. Load (mm)	Decrease in Deflection (%)
NR	25	94.43	-	22.63	-
	35	107.00	13.31	21.64	4.4
	45	115.64	22.46	20.27	10.4
CG	25	127.5	-	18	-
	35	156.71	23	7.28	59.6
	45	180.1	41.8	6.84	62
CGC	25	141.19	-	14.127	-
	35	173.88	23.15	10.83	23.3
	45	196.73	39.33	7.28	48.47

**Table 11:** Summary of the concrete compressive strength effects on the impact force.

Specimens	Compressive Strength (MPa)	Increase in Max. Impact Load (%) for Drop Height (mm)					Average Increasing Ratio (%)
		250	500	1000	1500	1900	
NR-I	35	45.25	65.44	2.53	3	9.78	25.2
	45	70.56	84.48	14.1	15.18	18.03	40.47
CG-I	35	13.31	7.76	27.14	28.27	16.02	18.5
	45	26.59	12.98	37.76	39.58	31.85	29.75
CGC-I	35	40.83	24.87	12.06	22.91	4.57	21.05
	45	75.83	48.87	27.96	38	13.54	40.84

**Table 12:** Summary of the concrete compressive strength effects on the deflection.

Specimens	Compressive Strength (MPa)	The Difference in Max. Deflection (%) for Drop Height (mm)					Average Difference Ratio (%)
		250	500	1000	1500	1900	
NR-I	35	−1.4	−0.9	27.27	0.89	14.59	9.03
	45	−14.9	22.18	36.48	43.5	36.56	30.72
CG-I	35	−16.7	6.32	39.03	29.15	5.46	19.34
	45	−28.1	47.15	58.38	52	12.14	39.56
CGC-I	35	−0.4	32.6	16.11	36.55	40.37	25.21
	45	−2.1	67.55	26.25	64	56.9	43.3

## Data Availability

The data presented in this study are available on request from the corresponding author.
